# The effect of a digital gamified breastfeeding counselling program on breastfeeding self-efficacy, breastfeeding success, and breast-related problems: a randomized controlled trial in Turkiye

**DOI:** 10.1093/her/cyag014

**Published:** 2026-04-29

**Authors:** Ozlem Ulku Bulut, Zehra Golbasi

**Affiliations:** Faculty of Health Sciences, Department of Midwifery, Lokman Hekim University, Sogutozu Mah., 2185, Cadde No: 20J, Cankaya, Ankara 06510, Turkiye; PhD Student, Department of Medical Education, Gazi University, Emniyet Mah., Bandirma Cad. No: 6/1, Yenimahalle, Ankara 06560, Turkiye; Faculty of Nursing, Lokman Hekim University, Sogutozu Mah., 2185, Cadde No: 20J, Cankaya, Ankara 06510, Turkiye

## Abstract

This single-site, pilot-scale randomized controlled study intended to evaluate the effects of a self-determination theory-informed (D6 gamification model-based) gamified breastfeeding counselling program on breastfeeding self-efficacy, breastfeeding success, and breast-related problems in postpartum participants. A pretest–posttest randomized controlled trial was conducted with 60 pregnant individuals recruited from a single university-affiliated private hospital in Türkiye, using a **pilot-scale** parallel group design. Participants were assigned to experimental (*n* = 30) and control (*n* = 30) groups by block randomization that was performed by an independent statistician. Due to the behavioural nature of the intervention, participant blinding was not feasible; however, outcome analyses were conducted by a blinded biostatistician. The intervention group received a digitally delivered gamified breastfeeding counselling program from gestational week 35 until week 2 postpartum, while the control group received routine prenatal and postnatal care. Data were obtained for validated measures of breastfeeding self-efficacy, breastfeeding success, and breast-related problems. Descriptive and inferential statistical analyses were conducted with SPSS 28.0 software. At week 2 postpartum, the mean breastfeeding self-efficacy scale-short form scores were significantly higher in the gamified counselling group than the control group (63.6 ± 6.2 versus 54.3 ± 8.4, *P* < .001, Cohen’s d = 1.26). Breastfeeding performance also favoured the intervention group, with higher infant breastfeeding assessment tool scores (8.6 ± 1.1 versus 7.3 ± 1.4, *P* < .001) and LATCH breastfeeding assessment scores (9.3 ± 0.7 versus 8.4 ± 1.1, *P* = .002). Furthermore, breast fullness severity that was assessed using a dichotomous self-reported outcome (present/absent) checklist in which higher scores indicate more severe fullness, was lower in the intervention group than the control group (2.5 ± 1.1 versus 4.0 ± 1.2, *P* < .001). Gamified breastfeeding counselling was effective in enhancing breastfeeding self-efficacy and breastfeeding success and reduced common breast-related problems. These findings provide preliminary evidence supporting the feasibility and short-term benefits of integrating gamification-based strategies into breastfeeding education, warranting larger multicenter studies with extended follow-up.

## Introduction

Breastfeeding is a key determinant of infant and maternal health outcomes, yet global adherence to recommended practices remains insufficient [1, 2]. Both the World Health Organization (WHO) and the United Nations Children’s Fund (UNICEF) advocate exclusive breastfeeding during the first 6 months postpartum, followed by continued breastfeeding alongside safe complementary foods for up to 2 years or longer [3, 4]. Despite these recommendations, adherence to ideal breastfeeding practices remains suboptimal globally and nationally. According to earlier WHO estimates, approximately 44% of infants younger than 6 months were exclusively breastfed worldwide; however, more recent UNICEF data indicate that this rate has increased to around 48% [5, 6]. In Türkiye, the exclusive breastfeeding rate remains approximately 41% [7]. Suboptimal breastfeeding is associated with an estimated 800 000 preventable child deaths annually [8], underscoring the need for effective and scalable breastfeeding support interventions. Breastfeeding self-efficacy, defined as a participant’s confidence in their ability to breastfeed, is a key determinant of breastfeeding initiation and continuation [9, 10]. Breastfeeding success refers to effective latch-on, adequate milk transfer, exclusive breastfeeding, and maternal satisfaction. Breast-related problems, including nipple pain, engorgement, mastitis, and perceived milk insufficiency are among the leading causes of early cessation of breastfeeding [5, 7, 11, 12]**.** Despite the availability of various breastfeeding education and counselling interventions, many programs still rely primarily on traditional face-to-face approaches. In a systematic review of 27 randomized controlled trials, Kim *et al.* [10] reported that supportive breastfeeding interventions (predominantly face-to-face and non-digital) significantly increased exclusive breastfeeding rates at 6 months, highlighting the limited evidence regarding digitally delivered and gamification-based approaches to breastfeeding support.

Since breastfeeding is a behaviour that requires sustained motivation and ongoing support, the effectiveness of education and counselling methods is crucial [12]. Gamification refers to incorporating game design elements (e.g. points, badges, levels, challenges, and feedback) into non-game settings to enhance motivation, engagement, and learning [13]. In health care and behavioural counselling, gamification has shown potential in improving knowledge retention, promoting behavioural activation, and supporting adherence to health-related interventions. Research suggests that customizing game design specifically to the individual can further enhance motivation and engagement, particularly in health education contexts [13–17].

The motivational elements in gamification are structured around learning objectives, duration, progression, and feedback mechanisms. Advancing through levels and receiving feedback can strengthen participants’ sense of competence while self-paced participation supports autonomy, both of which are essential for sustaining health behaviours, such as breastfeeding. The theoretical foundation of gamification is grounded in self-determination theory, which posits that autonomy, competence, and relatedness jointly foster intrinsic motivation [13–17]. In our study intervention, autonomy was supported with self-paced digital modules, competence *via* feedback and progressive challenges, and relatedness *via* ongoing counselling support. In a meta-analysis conducted by Kim and Castelli, game-based education programs across health domains enhanced intrinsic and extrinsic motivation and supported learning continuity [17].

Although various educational approaches exist to support breastfeeding, empirical evidence evaluating gamification-based breastfeeding counselling remains limited [8, 18]. This knowledge gap is particularly relevant in digitally accessible settings. Türkiye has a high internet penetration rate (95.5%) and widespread individual usage (87.1%) alongside high female literacy rates, indicating a favourable context for digital health interventions [19]. Therefore, integrating gamification into breastfeeding counselling could offer an innovative and contextually appropriate strategy to enhance participant engagement and breastfeeding-related outcomes.

The primary objective of this study was to evaluate the effect of gamified breastfeeding counselling on breastfeeding self-efficacy. Secondary objectives included assessing the effect of counselling on breastfeeding success and common breast-related problems. We hypothesized that participants receiving gamified breastfeeding counselling would show higher breastfeeding self-efficacy and success and fewer breast-related problems compared with those receiving routine care. Accordingly, this study examined the effects of gamified breastfeeding counselling delivered from gestational week 35 into the early postpartum period compared with standard practice.

## Methods

### Study design

This pretest–posttest parallel-group experimental study examined the effect of gamified breastfeeding counselling on breastfeeding self-efficacy, breastfeeding success, and breast problems. This randomized controlled trial was designed and reported in accordance with the CONSORT 2017 guidelines.

### Study group and sample calculation

The study included pregnant individuals between 32 and 34 weeks of gestation and was conducted at the Department of Obstetrics and Gynaecology, Etlik Lokman Hekim Hospital, Ankara, Türkiye, between January and July, 2023.

The participants attended the related hospital’s gynaecology outpatient clinics for pregnancy follow-up and controls. Those who agreed to participate were assigned to either experimental or control groups using the block randomization method.

A power analysis was performed to determine the required sample size. The statistical power of the test was calculated using G*Power 3.1 software. Based on a previous randomized controlled study by Selvi *et al.* (2021) [20], which reported a Cohen’s d effect size of 0.941 for differences in breastfeeding self-efficacy, the sample size was calculated using an effect size of 0.941, a power of 95%, and a significance of 0.05. According to this calculation, 52 participants (26 per group) were required (df = 50, t = 1.676). However, given that very large effect sizes might overestimate behavioural intervention effects, the sample size calculation should be interpreted as exploratory rather than confirmatory.

To account for any possible participant losses and based on similar research reporting up to 20% attrition, the sample size was increased to 60 participants, with at least 30 individuals per group [21].

**Figure 1:**
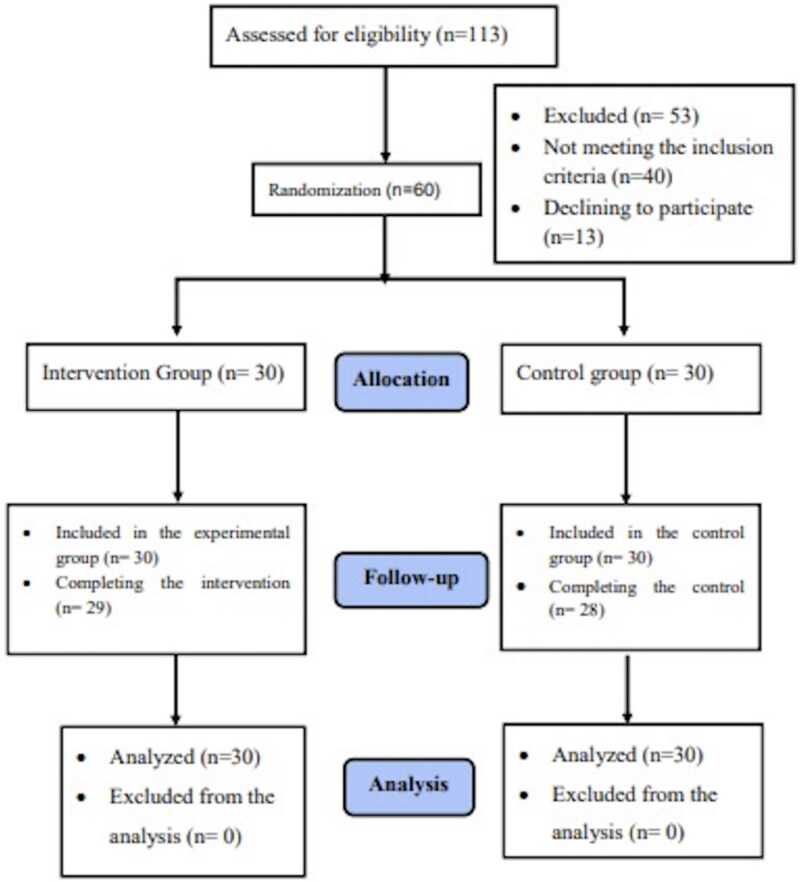
Consort flow diagram.

The gamified breastfeeding counselling program was prepared in accordance with the TIDieR Checklist (Intervention, Identification, and Replication Template), which facilitates better reporting of interventions [22, 23]. The trial was registered with clinicaltrials.gov (NCT 05435586) and a detailed protocol was published [24]. The study report was written based on CONSORT-2017 [25] criteria (Fig. 1).

### Data collection tools

#### Eligibility assessment form

This form was designed to assess participants’ eligibility for inclusion in the study and consists of ten items evaluating age, education level, gestational week, the presence of pregnancy risk factors, the history of infertility treatment, prior breastfeeding education, and smartphone ownership.

#### Sociodemographic and obstetric characteristics form

Developed based on the relevant literature [2, 10, 26], this 16-item form collects data on the participants’ sociodemographic (five items), obstetric and gynaecological history (eight items), and their knowledge about breastfeeding and skin-to-skin contact (three items).

#### Breastfeeding self-efficacy scale-short form (BSES-SF)

Developed by Dennis (2003) and adapted to Turkish by Tokat *et al.* (2010), this 14-item Likert-type scale measures maternal confidence in breastfeeding [26, 27]. Each item is scored from 1 (not at all confident) to 5 (always confident), with total scores ranging from 14 to 70. Higher scores indicate greater self-efficacy. In our study, Cronbach’s alpha was 0.892 (antenatal) and 0.906 (postnatal), indicating high internal consistency.

#### LATCH breastfeeding assessment tool

Developed by Jensen *et al.* (1994), LATCH evaluates five components of breastfeeding: latch, audible swallowing, type of nipple, comfort, and hold. Each item is scored from 0 to 2, yielding a total score between 0 and 10. Higher scores reflect better breastfeeding performance. The tool was validated for Turkish use by Yenal and Okumuş (2003) [28, 29].

#### Infant breastfeeding assessment tool (IBFAT)

The IBFAT was developed by Matthews in 1988 to assess early breastfeeding difficulties observed in full-term and healthy newborns during the first 4–5 days of life. The Turkish validity and reliability study was conducted in 2017 by Çelik, Odabaşı, and Demirci, who reported a Cronbach’s alpha coefficient of 0.92. In this study, the Cronbach’s alpha of the Turkish version was 0.73, indicating acceptable internal consistency [30, 31]. Both LATCH and IBFAT were used to capture complementary aspects of breastfeeding performance, with LATCH focusing on observable positioning and technique while IBFAT assessed early feeding behaviours and infant responses, thus providing a more comprehensive evaluation of breastfeeding success.

#### Postpartum information form

This nine item form was created to collect information regarding maternal and neonatal postpartum characteristics, including infant weight, length, and whether breastfeeding occurred within the first hour after birth [20, 21, 26].

#### Breastfeeding behaviour and breast problems assessment form

This form evaluates the mother’s breastfeeding frequency, exclusivity, duration, and common breast problems, such as pain, engorgement, nipple cracks, and mastitis. The form includes both yes and no and multiple choice items and was administered during both prenatal and postnatal periods [2, 20, 21, 31]. Breast fullness was assessed based on participant self-reporting using predefined checklist items rather than objective clinical measurement.

### Participants and procedure

Sixty primiparous participants between 32 and 34 weeks of gestation were randomly assigned to the experimental (*n* = 30) or control (*n* = 30) groups by an independent statistician, in accordance with CONSORT 2017 guidelines [25]. The inclusion criteria was that the participants had access to the relevant internet-enabled devices. The exclusion criteria included pregnancy risk factors (e.g. gestational diabetes or preeclampsia), planned caesarean delivery, or breastfeeding contraindications. These criteria were set to warrant sample homogeneity and minimize potential confounding factors that could influence breastfeeding outcomes. Participants who had caesarean deliveries were excluded because of the potential effect on early postpartum breastfeeding behaviours and maternal self-efficacy. While these criteria strengthened internal validity by reducing confounding factors, they also limit the generalizability of the findings to participants with uncomplicated vaginal births. The group assignments were communicated to the researcher on the phone by the statistician. Participant allocation was blinded until group assignment by ensuring that recruitment and baseline assessment were completed before the disclosure of allocation by the independent statistician.

The eligible participants completed the baseline assessments using the Preliminary Evaluation Form, the Form for Assessment of Descriptive and Obstetric Characteristics, and the Breastfeeding Self-Efficacy Scale–Antenatal Form [26]. The control group received routine prenatal and postnatal care. The experimental group received the gamified breastfeeding counselling program delivered *via* mobile devices from gestational weeks 35–37 and continued until the week 2 postpartum. Each session lasted 20–30 minutes. The program included interactive videos, scenario-based learning, quizzes, and motivational elements (e.g. badges and points).

All participants were assessed using validated tools during pregnancy and at weeks 2 and 8 postpartum, corresponding to early and mid-postpartum periods. These tools covered the Breastfeeding Self-Efficacy Scale (postnatal form), LATCH Scale, IBFAT Scale, Breastfeeding Behaviour and Problems Form, and the Postnatal Information Form. The study was completed with 57 participants (experimental *n* = 29 and control *n* = 28) and data were analysed based on the intention-to-treat (ITT) principle.

### Gamified breastfeeding counselling program

In the training program, ‘I Discover Breastfeeding’ and ‘My Breastfeeding Journey’ games were implemented during the 35–37 weeks of pregnancy and the first two weeks postpartum. The gamification process was formed based on the D6 model developed by Werbach and Hunter [32] and was enhanced with an escape room game format (Table 1). Escape room games are interactive and require players to solve puzzles and achieve the goal of escaping the room within a specified scenario. For instance, in one of the escape room scenarios, mothers were presented with a virtual hospital setting where their newborn showed signs of ineffective latch. To escape the room, they had to identify the incorrect latch position, choose the correct one from given options, and sequence the steps for initiating successful breastfeeding. Solving these challenges unlocked the next stage of the room, reinforcing key breastfeeding skills in an engaging and interactive way. The games were prepared as web-based activities *via* a Web 2.0 platform and were limited to approximately 20–30 minutes per session. A total of three sessions were held: once in the prenatal period and twice in the first two weeks after birth.

**Table 1:** Integration of the D6 model and gamified model into the designed program.

**D6 MODEL**	**Learning objective**	**Example**
**D1: Describe the objectives**	To ensure that participants have awareness, knowledge, and skills on breastfeeding self-efficacy, breastfeeding success, and breast problems.	Participants who completed the program received a certificate.
**D2: Define target behaviours**	To ensure that participants enter the program, learn breastfeeding techniques, and pass the levels.	Participants received certificates as they passed the levels.
**D3: Identify players**	To identify incremental behaviours aligned with participant’s motivations towards achieving their goals.	Participants successfully navigated escape rooms by assisting virtual participants. The design was tailored to the Philanthropist player type.
**D4: Design event loops**	Following a progression from simple to complex within the program, guiding participants on which instructions to follow, triggering behaviours, and addressing rewards for avoidance, *etc.*	The questions in the rooms progressed in difficulty as participants advanced.
**D5: Don’t forget the fun**	Shapes of badges/virtual items, animations, and rookie badges for those who pass the first level.	The virtual items were compared to the items in the baby’s room, and in the first episode, participants were given the ‘Rookie Mom’ certificate. Participants who completed all the chapters received the ‘Expert Mom’ certificate.
**D6: Use appropriate tools**	Using the appropriate mechanics and dynamics in the design of the program.	Components such as badges, levels, and virtual items were used.

The intervention was delivered digitally *via* smartphones or tablets. Game content was enriched with visual and auditory materials using AI-supported tools, including animations, interactive quizzes, voiceovers, and scenario-based tasks. Game elements, such as points, badges, progress tracking, and motivational feedback were incorporated to enhance engagement. The learning content was aligned with national breastfeeding education guidelines and standards, including topics, such as early initiation of breastfeeding, effective latch, feeding cues, milk production, and common challenges.

**Figure 2:**
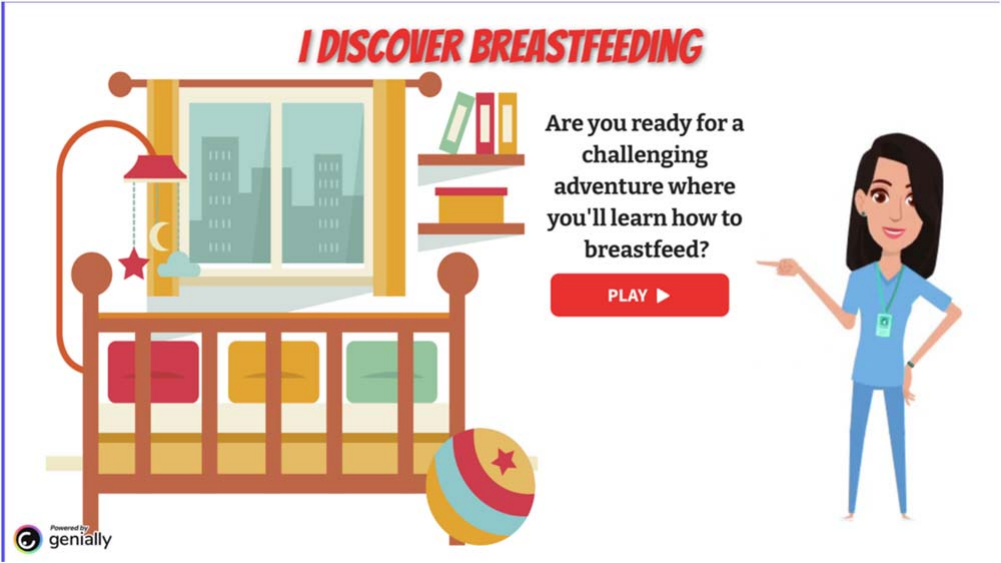
‘Discovering breastfeeding’ game login (genially.com). ^*^This image was designed by the researcher.

The participants’ access to the games was monitored *via* the platform’s usage logs, and completion of each session was tracked to assess adherence to the protocol. However, participant scores or in-game performance data were not linked to study outcomes (Fig. 2).

The game content was based on the national guidelines of the Turkish Ministry of Health for antenatal and postnatal breastfeeding education. The guidelines covered topics, such as the benefits of breastfeeding, effective latch technique, exclusive breastfeeding, management of breastfeeding problems, and continuation after returning to work. The control group also received standard breastfeeding counselling aligned with these guidelines in a conventional format as part of routine hospital care.

Before launching the intervention, a pilot test was conducted with 12 pregnant individuals to assess the technical performance and usability of the gamified program. Based on user feedback, improvements were made to content clarity, user interface, and navigation structure.

A detailed overview of the escape-room–based learning challenges included in the gamified breastfeeding counselling program is presented in Supplementary Table S1.

### Interventions applied to the control group

In this study, individuals in the control group received standard prenatal and postnatal care administered by the hospital. The interventions included routine antenatal follow-up visits every 4 weeks until 36 weeks, then weekly until birth, and postnatal care visits within the first 48 hours and weeks 2 and 8 postpartum. As part of the standard protocol in baby-friendly hospitals, basic breastfeeding counselling (e.g. breastfeeding positioning, feeding frequency, and signs of effective suckling) was provided. The care given followed national guidelines and standards, excluding the gamified breastfeeding guidance offered to the experimental group. The control group received the same breastfeeding education content based on national guidelines; however, this content was delivered in a conventional, non-gamified format without interactive or motivational elements.

After the final postpartum data collection at week 8, the gamified program was also offered to participants in the control group for ethical consistency. Although the game was originally designed for the prenatal and early postpartum periods, the postpartum modules included content on breastfeeding challenges, milk expression, and newborn care, making it beneficial for the mothers even after delivery.

### Data collection and follow-up

The participants in both the experimental and control groups were evaluated using the same set of validated instruments at three time points: baseline (gestation weeks 32–34), 2 weeks postpartum, and 8 weeks postpartum. The tools used the Breastfeeding Self-Efficacy Scale (antenatal and postnatal forms), LATCH Breastfeeding Assessment Tool, IBFAT Breastfeeding Assessment Scale, and Breastfeeding Behaviour and Breast Problems Assessment Form. The Postnatal Information Form was used at week 2 postpartum.

Fifty-seven of the 60 participants completed the study (intervention = 29 and control = 28). An ITT analysis approach was used and all participants were included in the final analyses. To address missing data, the last observation carried forward (LOCF) method was used to preserve both sample size and statistical power.

### Blinding

Due to the behavioural nature of the intervention, blinding of participants and researchers was not feasible, as both were aware of group allocation. This factor might have introduced performance bias, particularly given the novelty of the gamified intervention, which could have influenced participant responses in the experimental group. To minimize detection and analysis bias, all statistical analyses were performed by an independent biostatistician who was blinded to group assignments. Outcome assessors for IBFAT and LATCH were not blinded to group allocation, which could have introduced assessment bias; however, standardized scoring criteria were applied to reduce subjectivity.

### Ethical considerations

Ethical approval was granted by the Lokman Hekim University Scientific Research Ethics Committee (decision no: 2024/1–1). Informed written consent was obtained from all participants who met the inclusion criteria. Both groups received routine care in the hospital. At the end of the study, the control group were given the gamified breastfeeding counselling program to ensure equality.

### Data analysis

Parametric methods were used to analyse the data. Differences between the categorical variables in independent groups were analysed with Chi-squared and Fisher’s exact tests. The T-test was used to compare quantitative, continuous data between two independent groups. The dependent repeated measures ANOVA test was used to compare repeated measurements within the groups, and the Bonferroni test was used to determine from which group the difference originated.

All statistical analyses were performed with SPSS version 28.0 (IBM Corp., Armonk, NY). Descriptive statistics were presented as mean, standard deviation (SD), frequency, and percentage. Chi-squared and Fisher’s exact tests were used to compare categorical variables, while independent and paired samples T-tests were used to compare continuous variables. A *P* < .05 was considered statistically significant.

## Results

Of the 60 participants, three withdrew during follow-up (experimental = 1 and control = 2); therefore, the final analysis was conducted on 57 participants using the ITT principle. There were no significant differences between the groups in terms of sociodemographic and obstetric characteristics, such as age, education, employment status, income level, pregnancy planning, and intended breastfeeding duration, indicating a homogeneous distribution at baseline. Although identical proportions were observed for some of the categorical variables (e.g. educational level), verification of the dataset confirmed that these values resulted from random assignment rather than data duplication. Similarly, neonatal characteristics, including gestational week, delivery type, infant sex, neonatal intensive care unit need, early breastfeeding initiation, and average birthweight and length showed no significant differences between groups (*P* > .05).

At gestation week 37, the breastfeeding self-efficacy scores were significantly higher in the experimental group (mean = 58.30, SD = 5.8) compared with the control group (mean = 54.00, SD = 6.6; t[58] = 2.427, *P* = .018, d = 0.627; Table 2). This difference persisted at the week 8 postpartum, with the experimental group showing higher mean self-efficacy scores (mean = 59.70, SD = 6.5) than the control group (mean = 54.36, SD = 6.8; t[58] = 3.139, *P* = .003, d = 0.811). Repeated measures ANOVA revealed a significant group time interaction effect (F = 10.869, *P* = .002, η^2^ = 0.158), indicating that the increase in breastfeeding self-efficacy over time was significantly greater in the experimental group compared with the control group (Table 3).

**Table 2:** Maternal and neonatal characteristics of the intervention and control groups.

**Maternal characteristics**	**Intervention**		**Control**		**Total**		***P***
	***n***	**%**	***n***	**%**	***n***	**%**	
**Level of education**							
Primary school	0	0.0	1	3.3	1	1.7	X^2^ = 1.077 *P* = .584
Lower secondary school	7	23.3	6	20.0	13	21.7	
High school or above	23	76.7	23	76.7	46	76.7	
**Employment status**							
Not employed	11	36.7	16	53.3	27	45.0	X^2^ = 1.684 *P* = .150
Employed	19	63.3	14	46.7	33	55.0	
**Perceived income status**							
Income less than expenses	1	3.3	2	6.7	3	5.0	X^2^ = 1.117 *P* = .572
Income equal to expenses	21	70.0	23	76.7	44	73.3	
Income more than expenses	8	26.7	5	16.7	13	21.7	
**Family type**							
Nuclear family	29	96.7	28	93.3	57	95.0	X^2^ = 1.018 *P* = .601
Extended family	1	3.3	1	3.3	2	3.3	
Single-parent family	0	0.0	1	3.3	1	1.7	
**Gestational week**							
32nd week	5	16.7	1	3.3	6	10.0	X^2^ = 2.963 *P* = .227
33rd week	6	20.0	7	23.3	13	21.7	
34th week	19	63.3	22	73.3	41	68.3	
**Pregnancy planning status**							
Planned	25	83.3	23	76.7	48	80.0	X^2^ = 0.417 *P* = .374
Unplanned	5	16.7	7	23.3	12	20.0	
**Intended breastfeeding duration after birth**							
0–12 months	5	16.7	7	23.3	12	20.0	X^2^ = 1.154 *P* = .561
0–24 months	21	70.0	17	56.7	38	63.3	
0–36 months	4	13.3	6	20.0	10	16.7	
**Birth and Postpartum Characteristics**							
**Gestational week at birth**							
37 38 39 40 41	6	20.0	7	23.3	13	21.7	X^2^ = 6.377 *P* = .173
	9	30.0	9	30.0	18	30.0	
	8	26.7	12	40.0	20	33.3	
	7	23.3	1	3.3	8	13.3	
	0	0.0	1	3.3	1	1.7	
**Mode of delivery**							
Vaginal birth	14	46.7	14	46.7	28	46.7	X^2^ = 0.000 *P* = .602
Caesarean section	16	53.3	16	53.3	32	53.3	
**Infant Sex**							
Female	13	43.3	11	36.7	24	40.0	X^2^ = 0.278 *P* = .396
Male	17	56.7	19	63.3	36	60.0	
**Admission to neonatal intensive care unit (NICU)**							
Yes No	3	10.0	4	13.3	7	11.7	X^2^ = 0.162 *P* = .500
	27	90.0	26	86.7	53	88.3	
**Breastfeeding within the first hour after birth**							
Yes No	26	86.7	25	83.3	51	85.0	X^2^ = 0.131 *P* = .500
	4	13.3	5	16.7	9	15.0	

	**Mean**	**SD**	**Mean**	**SD**	**Mean**	**SD**	***P***
**Infant birth weight**	3271.83	430.08	3288.50	426.25	3280.17	424.61	.881
**Infant birth length**	50.57	3.30	49.23	1.56	49.90	2.65	.051


*Chi-square analysis; Independent samples t-test.*

**Table 3:** The mean BSES-SF scores of participants in the experimental and control groups.

**Groups**	**Intervention (*n* = 30)**		**Control (*n* = 30)**		**t** ^**a**^	***P***	**Lower %95 CI**	**Upper %95 CI**	**d**
	**Mean**	**Sd**	**Mean**	**Sd**					
BSES-SF Pretest	48.36	6.85	47.70	11.76	.268	.790	−4.308	5.641	
BSES-SF Interim Test (Week 37)	58.30	7.55	54.00	6.09	2.427	**.018**	0.754	7.846	0.627
BSES-SF Postnatal Form Posttest	59.70	6.73	54.36	6.41	3.139	**.003**	1.932	8.734	0.811
**F** ^**b**^	26.824		5.497						
***P***	**.000**		**.011**						
**Bonferroni**	**1 < 2,3**		**1 < 2,3**						
**η** ^**2**^	0.481		0.159						
Group*Time	**F = 10.869; *P* = 0.002; η** ^**2**^ **= 0.158**								

a Independent Groups t-Test.

b Repeated Measures Anova Test

**Table 4:** Comparison of IBFAT and LATCH scores and breastfeeding status between experimental and control groups.

**Groups**	**Intervention (*n* = 30)**		**Control (*n* = 30)**		**t** ^**a**^	**sd**	***P***	**Lower %95 CI**	**Upper %95 CI**	**d**
	**Mean**	**Sd**	**Mean**	**Sd**						
IBFAT	8.43	1.83	7.20	2.36	2.255	58	**.028**	0.138	2.327	0.582
LATCH	8.06	1.43	7.00	1.85	2.488	58	**.016**	0.208	1.925	0.643

a Independent Groups T-Test. X^2^: Chi-Square Analysis

Infants in the experimental group showed more effective feeding behaviours. Specifically, 40.0% were categorized as effectively feeding and 43.3% as successfully suckling, while in the control group, these rates were 26.7% and 13.3%, respectively. The proportion of short-interval suckling was notably lower in the experimental group (16.7%) than in the control group (60.0%), with a significant difference observed (χ^2^ = 12.913, *P* = .002; Table 5).

**Table 5:** IBFAT breastfeeding status of the experimental and control groups.

**IBFAT**	**Intervention**		**Control**		**Total**		***P***
	***n***	**%**	***n***	**%**	***n***	**%**	
Effectively Feeding Babies	12	40.0	8	26.7	20	33.3	X^2^ = 12.913 ***P* = .002**
Successfully Suckling Babies	13	43.3	4	13.3	17	28.3	
Suckling Babies with Short Intervals	5	16.7	18	60.0	23	38.3	
Chi-Square Analysis							

Regarding breastfeeding problems, there were no significant differences between the groups at week 2 postpartum in terms of nipple cracks, redness, fever, difficulty latching, or use of complementary feeding (*P* > .05; Table 5). At week 8 postpartum, these outcomes remained comparable between groups with the exception of breast fullness, which was significantly less frequent in the experimental group (χ^2^ = 5.192, *P* = .026). Breast fullness was assessed as a dichotomous self-reported outcome (present or absent).

These findings suggest that the gamified breastfeeding counselling program significantly improved antenatal and postnatal breastfeeding self-efficacy and success, while also reducing breast fullness complaints in the later postpartum period (Table 6).

**Table 6:** Comparison of breast problems in the 2nd and 8th weeks postpartum between experimental and control groups.

**Groups**	**Intervention**				**Control**				***P*** **2nd week postpartum**	***P*** **8nd week postpartum**
	**2nd week postpartum**		**8th week** **postpartum**		**2nd week postpartum**		**8th week postpartum**			
	***n***	**%**	***n***	**%**	***n***	**%**	***n***	**%**		
**Complementary feeding other than breast milk**										
Yes	1	3.3	3	10.0	1	3.3	4	13.3	X^2^ = 0.000 *P* = .754	X^2^ = 0.162 *P* = .500
No	29	96.7	27	90.0	29	96.7	26	86.7		
**Difficulty in latching on**										
Yes	9	30.0	11	36.7	6	20.0	10	33.3	X^2^ = 0.800 *P* = .276	X^2^ = 0.073 *P* = .500
No	21	70.0	19	63.3	24	80.0	20	66.7		
**Cracked Nipple/Sores**										
Yes	11	36.7	3	10.0	7	23.3	4	13.3	X^2^ = 1.270 *P* = .199	X^2^ = 0.162 *P* = .500
No	19	63.3	27	90.0	23	76.7	26	86.7		
**Breast Fullness**										
Yes	4	13.3	1	3.3	9	30.0	7	23.3	X^2^ = 2.455 *P* = .105	X^2^ = 5.192 ***P* = .026**
No	26	86.7	29	96.7	21	70.0	23	76.7		
**Breast Abscess**										
Yes	1	3.3	0	0.0	2	6.7	1	3.3	X^2^ = 0.351 *P* = .500	X^2^ = 1.017 *P* = .500
No	29	96.7	30	100.0	28	93.3	29	96.7		

X^2^: Chi-Square Analysis.

At week 2 postpartum, the experimental group had significantly higher IBFAT scores (mean = 8.43, SD = 1.1) compared with the control group (mean = 7.20, SD = 1.2; t[58] = 2.255, *P* = .028, d = 0.582). Likewise, LATCH scores were higher in the experimental group (mean = 8.06, SD = 0.9) than in the control group (mean = 7.00, SD = 1.1; t[58] = 2.488, *P* = .016, d = 0.643; Table 4).

## Discussion

This study evaluated the effectiveness of a gamified breastfeeding counselling program on breastfeeding self-efficacy, breastfeeding success, and breast-related problems. The findings indicate that participants who received gamified counselling had higher breastfeeding self-efficacy scores and more favourable breastfeeding assessment outcomes compared with those receiving routine care. Importantly, these results should be interpreted as associations observed within the context of the study design rather than evidence of causality.

Initially, there were no significant differences between the groups in baseline characteristics, such as the sociodemographic, obstetric, and antenatal breastfeeding self-efficacy scores, indicating adequate group homogeneity at study entry. However, after the antenatal module of the intervention (week 37), the experimental group had significantly higher breastfeeding self-efficacy scores compared with the control group. This difference was significant and practically meaningful, with a moderate effect size. After completion of the postnatal module, breastfeeding self-efficacy scores at week 8 postpartum remained significantly higher in the experimental group, with a large effect size (d = 0.811), indicating a substantial improvement in perceived breastfeeding confidence.

Although significance alone does not necessarily imply clinical relevance, the moderate-to-large effect sizes observed in this study suggest meaningful implications for breastfeeding support, particularly when interpreted alongside behavioural indicators, such as successful latching and breastfeeding continuation. Previous studies similarly underscore the effects of breastfeeding support interventions. For instance, Sihombing *et al.* [2] showed that a card game-based gamified education improved breastfeeding self-efficacy, knowledge, and success. In their study, breastfeeding self-efficacy scores increased by approximately 6–8 points following the intervention, indicating a magnitude of change comparable to the improvements observed in our study. Similarly, a single-blind randomized controlled trial by Chehreh *et al.* [21] showed that a breastfeeding support program increased breastfeeding self-efficacy. These findings align with other studies that have shown various breastfeeding education methods positively affecting breastfeeding self-efficacy [2, 10, 31–35]. Distinct from conventional counselling approaches, the gamified breastfeeding counselling program was designed to make the breastfeeding process more interactive and engaging, which might have facilitated greater internalization of breastfeeding-related skills and confidence. Of note, the observed improvements might partially reflect increased attention and novelty effects (Hawthorne effect) associated with participation in an interactive intervention, rather than the gamification components alone.

In our study, the experimental group showed significantly higher breastfeeding success, as reflected by both LATCH and IBFAT scores at week 2 postpartum. The effect sizes (d = 0.643 for LATCH and d = 0.582 for IBFAT) support the practical significance of these outcomes. Furthermore, the proportion of infants who breastfed effectively or successfully suckled was higher in the experimental group. These results align with previous studies that have reported that prenatal and postnatal breastfeeding education enhances breastfeeding success [36–39]. For instance, systematic reviews from low-income and middle-income settings have confirmed that support programs improve early initiation and continuation of exclusive breastfeeding [37].

Regarding exclusive breastfeeding behaviour, our study found no significant difference between groups at the weeks 2 and 8 postpartum, with only 3% of participants in each group reporting the use of complementary food. This finding might reflect a ceiling effect associated with high baseline awareness and effective routine breastfeeding education rather than an absence of intervention effect. This finding diverges from previous research where higher rates of early complementary feeding was observed, such as in the study by Özsoy and Dündar reporting 30% prevalence due to insufficient postnatal education [40]. The low prevalence in both groups might reflect increased awareness due to broader public health messaging or the comprehensive antenatal education received.

For breast-related problems, while no significant differences were observed at week 2 postpartum, the intervention group reported significantly fewer instances of breast fullness by the week 8. This finding supports earlier reports indicating that breast fullness often peaks during the first postpartum month and can persist in the absence of adequate breastfeeding support [41, 42]. Therefore, the reduction in breast fullness observed among participants receiving gamified counselling could reflect improved milk management and breastfeeding practices facilitated by the intervention.

Breast fullness, although sometimes interpreted as an indicator of sufficient milk supply, is frequently linked to engorgement and discomfort if it persists beyond the early postpartum period. In our study, the control group had a significantly higher occurrence of breast fullness at week 8 postpartum (X^2^ = 5.192, *P* = .026), which could reflect unresolved engorgement or inadequate milk removal. Such conditions can contribute to maternal discomfort, increase the risk of mastitis, and reduce breastfeeding satisfaction, potentially undermining the continuation of breastfeeding. Conversely, the lower prevalence of breast fullness in the intervention group suggests a potential protective role of gamified counselling in supporting effective breastfeeding management. Overall, our results indicate that gamified breastfeeding counselling programs represents a promising and contextually relevant approach for enhancing breastfeeding self-efficacy, improving early breastfeeding success, and reducing selected breast-related problems. Nevertheless, further studies with larger samples and longer follow-up periods are warranted to evaluate long-term clinical outcomes and broader implementation feasibility.

### Limitations of the study

Several limitations of this study should be acknowledged. Blinding during data collection was not feasible as both participants and researchers were aware of group assignments, which could have introduced performance and assessment bias. Although statistical analyses were conducted by a blinded biostatistician, the absence of full blinding remains an inherent limitation of behavioural intervention studies. The study was conducted in the premises of a private hospital with participants predominantly from higher socioeconomic backgrounds, which might restrict the generalizability of the findings. Additionally, the sample size calculation was based on an effect size (Cohen’s d = 0.941) derived from previous literature, which may be considered optimistic for behavioural interventions. This reliance on high effect size parameters, combined with the pilot-scale nature of the study, means the results should be viewed as exploratory rather than confirmatory, as they may overestimate the actual intervention effects. Furthermore, participants who lacked access to digital devices or adequate digital literacy were excluded, potentially limiting the feasibility of implementing this intervention in low-resource or underserved settings. To assure internal validity, the study excluded participants with high-risk pregnancies, planned caesarean sections, and contraindications to breastfeeding. While this strategy reduced clinical heterogeneity and potential confounding, it also narrowed the applicability of the findings to healthier maternal populations, and the results might not be directly transferable to participants with more complex obstetric profiles. Although participants’ use of educational modules and login frequency were digitally tracked, these engagement metrics were not analysed or reported as outcome measures. As a result, the degree of participant interaction with the intervention content could not be evaluated in relation to study outcomes. Future studies would benefit from integrating qualitative feedback and engagement analytics to better understand user experience and intervention mechanisms.

Missing outcome data were handled using the LOCF method, which may introduce potential bias by assuming stability of outcomes over time. Therefore, the results should be interpreted with caution, and future studies with larger samples and more advanced missing-data techniques are recommended.

This study followed its participants from pregnancy and continued monitoring through to week 8 postpartum, whereas WHO recommends exclusive breastfeeding for the first 6 months postpartum. Thus, the 8-week follow-up period limits the ability to draw conclusions about the long-term sustainability of exclusive breastfeeding behaviours.

Despite these limitations, the study was strengthened by its structured and theory-informed intervention design, the use of validated measurement tools, and random assignment of participants, all of which are factors that contribute to the robustness of internal validity and support the reliability of the observed associations.

## Conclusion

This study showed that participation in a gamified breastfeeding counselling program was associated with higher breastfeeding self-efficacy and improved early breastfeeding success compared with routine care. Participants in the intervention group showed moderate-to-large improvements in breastfeeding self-efficacy across the antenatal and early postnatal periods, alongside higher LATCH and IBFAT scores at week 2 postpartum, indicating more effective breastfeeding behaviours. In addition, a lower prevalence of breast fullness was observed in the intervention group at week 8 postpartum, suggesting a potential short-term benefit of gamified counselling for milk management and maternal comfort.

Although no significant differences were observed between groups in exclusive breastfeeding rates during the early postpartum period, the uniformly high rates in both groups might reflect a ceiling effect related to comprehensive routine care and high baseline breastfeeding awareness. Taken together, these findings provide preliminary evidence that gamification-based counselling could complement conventional breastfeeding education by supporting maternal confidence and early breastfeeding performance. However, given the short follow-up period, single-centre design, and restricted sample characteristics, further large-scale, multicenter studies with extended follow-up are required before drawing conclusions regarding long-term effectiveness, scalability, or integration into routine maternal health services.

## Supplementary Material

Supplementary_Material_cyag014

## Data Availability

The data supporting the findings of this study are not publicly available due to ethical and privacy considerations. However, anonymized data may be made available from the corresponding author upon reasonable request and with appropriate ethical approval.
