# Supplementary material for: The effect of a digital gamified breastfeeding counselling program on breastfeeding self-efficacy, breastfeeding success, and breast-related problems: a randomized controlled trial in Turkiye
Source: Health Educ Res. 2026 Apr 29;41(3):cyag014. doi: 10.1093/her/cyag014 (PMC13127138; doi:10.1093/her/cyag014)
Supplement: Supplementary_Material_cyag014 [file supplementary_material_cyag014.doc]

**Supplementary Table S1. Structure of the Gamified Breastfeeding Counseling Program and Escape-Room Challenges**

| **Game Module** | **Period** | **Scenario Theme** | **Learning Objective** | **Challenge Task** | **Gamification Elements** |
| --- | --- | --- | --- | --- | --- |
| **I Discover Breastfeeding** | 35–36 gestational weeks | Importance of breastfeeding | Understanding the health benefits of breastfeeding for mother and infant | Participants identify correct statements about breastfeeding benefits to unlock the first room | Points, progress bar |
| **I Discover Breastfeeding** | 36 gestational weeks | Breastfeeding physiology | Understanding milk production and hormonal regulation | Participants arrange the correct sequence of physiological processes involved in milk production | Puzzle challenge, feedback |
| **I Discover Breastfeeding** | 37 gestational weeks | Early initiation of breastfeeding | Recognizing newborn feeding cues and early breastfeeding behaviors | Participants match newborn hunger cues with appropriate maternal responses | Badges, level progression |
| **My Breastfeeding Journey** | Early postpartum | Correct latch technique | Identifying correct latch and positioning | Participants analyze a scenario in which a newborn shows signs of ineffective latch and select the correct breastfeeding position | Escape-room puzzle, immediate feedback |
| **My Breastfeeding Journey** | Early postpartum | Managing breastfeeding problems | Recognizing and managing common breastfeeding problems such as nipple pain or engorgement | Participants select appropriate strategies for managing breastfeeding difficulties | Points, challenge completion |
| **My Breastfeeding Journey** | Early postpartum | Maintaining breastfeeding | Supporting exclusive breastfeeding and milk continuity | Participants organize the correct steps for maintaining milk production and breastfeeding continuation | Level completion, digital rewards |
